# Tumor suppressive *microRNA-218* inhibits cancer cell migration and invasion by targeting focal adhesion pathways in cervical squamous cell carcinoma

**DOI:** 10.3892/ijo.2013.1851

**Published:** 2013-03-07

**Authors:** NORIKO YAMAMOTO, TAKASHI KINOSHITA, NIJIRO NOHATA, TOSHIHIKO ITESAKO, HIROFUMI YOSHINO, HIDEKI ENOKIDA, MASAYUKI NAKAGAWA, MAKIO SHOZU, NAOHIKO SEKI

**Affiliations:** 1 Departments of Functional Genomics, Chiba University Graduate School of Medicine, Chiba;; 2 Obstetrics and Gynecology Medicine, Chiba University Graduate School of Medicine, Chiba;; 3 Department of Urology, Graduate School of Medical and Dental Sciences, Kagoshima University, Kagoshima, Japan

**Keywords:** microRNA, miR-218, tumor suppressor, cervical cancer, LAMB3, focal adhesion

## Abstract

Cervical cancer is one of the most common cancers in women. More than 275,100 women die from cervical cancer each year. Cervical squamous cell carcinoma (cervical SCC), one of the most frequent types of cervical cancers, is associated with high-risk human papilloma virus (HPV), although HPV infection alone may not be enough to induce malignant transformation. MicroRNAs (miRNAs), a class of small non-coding RNAs, regulate protein-coding gene expression by repressing translation or cleaving RNA transcripts in a sequence-specific manner. A growing body of evidence suggests that miRNAs contribute to cervical SCC progression, development and metastasis. miRNA expression signatures in SCC (hypopharyngeal SCC and esophageal SCC) revealed that *miR-218* expression was significantly reduced in cancer tissues compared with adjacent non-cancerous epithelium, suggesting that *miR-218* is a candidate tumor suppressor. The aim of this study was to investigate the functional significance of *miR-218* in cervical SCC and to identify novel *miR-218*-mediated cancer pathways in cervical SCC. Restoration of *miR-218* significantly inhibited cancer cell migration and invasion in both HPV-positive and HPV-negative cervical SCC cell lines. These data indicated that *miR-218* acts as a tumor suppressor in cervical SCC. Our *in silico* analysis showed that *miR-218* appeared to be an important modulator of tumor cell processes through suppression of many targets, particularly those involved in focal adhesion signaling pathways. Gene expression data indicated that *LAMB3*, a laminin protein known to influence cell differentiation, migration, adhesion, proliferation and survival, was upregulated in cervical SCC clinical specimens, and silencing studies demonstrated that *LAMB3* functioned as an oncogene in cervical SCC. The identification of novel tumor-suppressive *miR-218*-mediated molecular pathways has provided new insights into cervical SCC oncogenesis and metastasis.

## Introduction

Cervical cancer is one of the most common cancers in women. It has been estimated that more than 529,800 new cases will be diagnosed each year, and approximately 275,100 women worldwide will die of cervical cancer each year (1). Cervical squamous cell carcinoma (cervical SCC) is one of the most frequent types of cervical cancers, accounting for 80–90% of cervical cancers, and the most important risk factor for cervical SCC is persistent human papilloma virus (HPV) infection (2). Epidemiological studies have indicated that more than 99% of patients with cervical SCC are positive for high-risk HPV (HPV16, HPV18 and HPV31) (3,4). The high-risk HPVs contain oncoproteins, i.e., E6 and E7, which contribute to oncogenesis of cervical SCC by silencing the tumor-suppressive p53 and Rb proteins (5–8). The molecular mechanisms of cervical SCC initiation, development and metastasis have not yet been fully elucidated. Therefore, an increased understanding of the molecular targets and pathways of cervical SCC progression and metastasis is necessary, preferably using latest approaches in genomic analysis, including non-coding RNA studies.

RNA can be divided into 2 categories: protein-coding RNA and non-coding RNA (ncRNA). It is important to examine the functions of ncRNAs and their association with human disease, including cancer. MicroRNAs (miRNAs) are endogenous small ncRNA molecules (19–22 bases in length) that regulate protein-coding gene expression by repressing translation or cleaving RNA transcripts in a sequence-specific manner (9). A growing body of evidence suggests that miRNAs are aberrantly expressed in many human cancers and that they play significant roles in the initiation, development and metastasis of these cancers (10). Some highly expressed miRNAs can function as oncogenes by repressing tumor suppressors, whereas low-level miRNAs can function as tumor suppressors by negatively regulating oncogenes (11).

We previously performed miRNA expression signature analysis of hypopharyngeal, maxillary sinus, esophageal and lung SCCs, in addition to bladder cancer and renal cell carcinoma; these studies indicated that *miR-218* was significantly reduced in cancer tissues compared with adjacent non-cancerous tissues, suggesting that *miR-218* is a candidate tumor-suppressive miRNA in human cancers (12–18). The results of past functional studies of *miR-218* in various cancers indicated that *miR-218* inhibits cancer cell proliferation and invasion through targeting oncogenic genes (19–23). Interestingly, *miR-218* was underexpressed in HPV-positive cell lines, cervical lesions, and cancer tissues containing HPV16 DNA, as compared to both C-33A cells and normal cervical tissues (24).

The aim of the study was to investigate the functional significance of *miR-218* in both HPV-positive and -negative cell lines and to identify the molecular pathways mediating *miR-218* in cervical SCC cells. Genome-wide gene expression data for *miR-218* and *in silico* database analyses showed that the focal adhesion pathway was a promising *miR-218* target pathway. The laminins *LAMB3* and *LAMC1* are an important and biologically active part of the basal lamina, influencing cell differentiation, migration, adhesion, proliferation and survival. In this study, we focused on *LAMB3* and investigation of the functional significance of this gene in cervical SCC. The novel tumor-suppressive *miR-218*-mediated cancer pathways identified herein provide new insights into the potential mechanisms of cervical SCC oncogenesis and metastasis.

## Materials and methods

### Clinical specimens

A total of 18 primary cervical SCC specimens and 11 non-cancerous specimens were collected from patients who had undergone surgical treatment at Chiba University Hospital. The samples were processed and stored in RNAlater (Qiagen, Valencia, CA, USA) at −20°C until RNA extraction. Patient information is summarized in Table I. Our study was approved by the Bioethics Committee of Chiba University; prior written informed consent and approval was given by each patient.

### Cervical SCC cell lines

CaSki (HPV16-positive) and ME180 (HPV39-positive) cells were grown in RPMI-1640 medium supplemented with 10% fetal bovine serum. HeLa (HPV18-positive) cells were grown in E-MEM medium supplemented with 10% fetal calf serum, and Yumoto (HPV-negative) cells were grown in E-MEM medium supplemented with 10% fetal bovine serum. All cells were cultured in a humidified atmosphere containing 5% CO_2_ at 37°C.

### RNA isolation

Total RNA was isolated using TRIzol Reagent (Invitrogen, Carlsbad, CA, USA) according to the manufacturer’s protocol. RNA concentration was determined spectrophotometrically. RNA quality was confirmed using a NanoDrop 1000 Spectrophotometer (Thermo Fisher Scientific, Wilmington, DE, USA).

### DNA isolation and HPV status

Genomic DNA was extracted by QIAamp DNA mini kit (Qiagen, Venlo, The Netherlands). Samples without HPV infection were determined by PCR amplification using the L1 consensus primers MY09 and MY11 as described previously (25). HPV-positive samples were analyzed to determine the presence of DNA for HPV16 and HPV18. We designed type-specific real-time PCR primers for the E6 and E7 regions of HPV16 and HPV18 (Table II), and real-time PCR was performed using a LightCyclerNano PCR System according to the manufacturer’s protocol.

### Quantitative real-time RT-PCR

Stem-loop RT-PCR (TaqMan MicroRNA assays; P/N: 000521 for *miR-218*; Applied Biosystems, Foster City, CA, USA) was used to quantify miRNAs according to earlier published conditions (13). To normalize the data for quantification of *miR-218*, we used *RNU48* (assay ID: 001006; Applied Biosystems). TaqMan probes and primers for *LAMB3* (P/N: Hs00165078_m1) and *GAPDH* (P/N: Hs02758991_g1) were obtained from Applied Biosystems. Primers for *ACTB* (P/N: ACTB 533F 37546-020, ACTB 653R 37546-021) were obtained from Sigma genetics. We used the ΔΔCt method to calculate the fold-change.

### Mature miRNA and siRNA transfections

Cervical SCC cell lines were transfected with Lipofectamine RNAiMAX transfection reagent (Invitrogen) and Opti-MEM (Invitrogen) with 10 nM mature miRNA or siRNA molecules. The following RNA species were used in this study: mature miRNA, Pre-miR miRNA Precursor (hsa-miR-218; Applied Biosystems, P/N: AM17100), negative control miRNA (Applied Biosystems, P/N: AM17111), small-interfering RNA (Silencer Select, Applied Biosystems, si-LAMB3, P/N: s8075 and s8076), and negative control siRNA (Stealth RNAi Negative Control Medium GC Duplex, Invitrogen, P/N: 12935-300). Cells were seeded in 10-cm dishes for protein extraction (8×10^5^ cells per dish), 24-well plates for mRNA extraction and Matrigel invasion assays (5×10^4^ cells per well), and 96-well plates for XTT assays (ME180: 3,000 cells per well; CaSki and HeLa: 4,000 cells per well; and Yumoto: 2×10^4^ cells per well).

### Cell proliferation, migration, and invasion assays

Cell proliferation was determined using an XTT assay (Roche Applied Science, Tokyo, Japan) according to the manufacturer’s instructions. Cell migration assays were performed using modified Boyden Chambers (Transwells, Costar #3422, Corning Incorporated, Corning, NY, USA) containing uncoated Transwell polycarbonate membrane filters with 8-*μ*m pores in 24-well tissue culture plates. Cells were transfected with 10 nM miRNA by reverse transfection and plated in 10-cm dishes at 8×10^5^ cells. After 48 h, 1×10^5^ cells were added to the upper chamber of each migration well and were allowed to migrate for 48 h. After gentle removal of the non-migratory cells from the filter surface of the upper chamber, the cells that migrated to the lower side were fixed and stained with Diff-Quick (Sysmex Corporation, Kobe, Japan). The number of cells migrating to the lower surface was determined microscopically by counting 4 areas of constant size per well. A cell invasion assay was carried out using modified Boyden chambers containing transwell-precoated Matrigel membrane filter inserts with 8-*μ*m pores in 24-well tissue culture plates at 1×10^5^ cells per well (BD Biosciences, USA). All experiments were performed in duplicate.

### Pathway analysis and expression data of putative miR-218 target genes

To obtain putative *miR-218* regulated genes, we adopted a TargetScan database searching method (http://www.targetscan.org). To identify molecular targets and signaling pathways regulated by *miR-218*, *in silico* and gene expression data were analyzed in the Kyoto Encyclopedia of Genes and Genomics (KEGG) pathway (http://www.genome.jp/kegg/pathway.html) categories using the GeneCodis program (http://genecodis.cnb.csic.es/). In this study, we focused on the focal adhesion pathway, which included 48 genes. Gene expression data were applied using the GEO database (accession no. GSE6791).

### Western blot analysis

Cells were harvested and lysed 72 h after transfection. Each cell lysate (50 *μ*g of protein) was separated using Mini-PROTEAN TGX gels (Bio-Rad, Hercules, CA, USA), followed by subsequent transfer to PVDF membranes. Immunoblot analysis was performed with polyclonal anti-LAMB3 antibodies (HPA008069; Sigma-Aldrich, St. Louis, MO, USA). Anti-GAPDH antibodies (ab8245; Abcam, Cambridge, UK) were used as an internal control. The membrane was washed and incubated with anti-rabbit IgG, HRP-linked antibodies (#7074; Cell Signaling Technology, USA). Complexes were visualized with an Immun-Star™ WesternC Chemiluminescence Kit (Bio-Rad), and the expression levels of these proteins were evaluated by ImageJ software (ver.1.44; http://rsbweb.nih.gov/ij/index.html).

### Plasmid construction and dual-luciferase reporter assays

Partial sequences of the *LAMB3* 3′ untranslated region (3′UTR) that contain the *miR-218* target site (ggcatgccattgaaactaagagctctcaagtcaaggaagctgggctgggcagtatcccccgcctttagttctccactggggaggaatcctggaccaagcacaaaaacttaacaaaagtgatgtaaaaatgaaaagccaaataaaatctttggaaaagagcctggaggttc) were inserted between the *Xho*I and *Pme*I restriction sites in the 3′UTR of the *hRluc* gene in the psiCHECK-2 vector (Promega, Madison, WI, USA). CaSki was then transfected with 5 ng vector, 10 nM mature miRNA. Firefly and *Renilla* luciferase activities in cell lysates were determined using a dual-luciferase assay system (E1910; Promega). Normalized data were calculated as the quotient of *Renilla*/firefly luciferase activities.

### Statistical analysis

The relationships between 2 variables and numerical values were analyzed using the Mann-Whitney U test, and the relationships between 3 variables and the numerical values were analyzed using the Bonferroni-adjusted Mann-Whitney U test. Expert Stat View analysis software (ver. 4; SAS Institute Inc., Cary, NC, USA) was used in both analyses. In the comparison of 3 variables, a non-adjusted statistical level of significance of P<0.05 corresponded to the Bonferroni-adjusted level of P<0.0167.

## Results

### Expression levels of miR-218 in cervical SCC clinical specimens and cell lines

**Figure 1:**
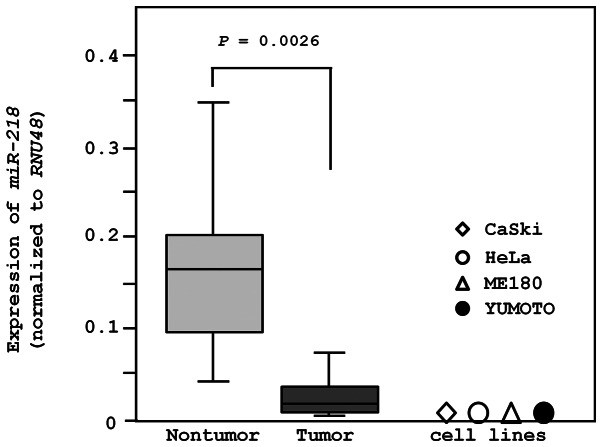
Expression of *miR-218* in cervical-SCC clinical specimens and cell lines. Expression of *miR-218* in cervical-SCC tumor tissues, nontumor tissues, and cell lines as determined by qRT-PCR. *RNU48* was used as an internal control.

The expression of *miR-218* was significantly lower in clinical cervical SCC specimens (n=18; 0.043±0.077) than in non-cancerous specimens (n=11; 0.153±0.110, P=0.0026; Fig. 1). We also evaluated the expression of *miR-218* in cervical cancer cell lines. *miR-218* expression levels in CaSki (HPV16-positive), ME180 (HPV39-positive), HeLa (HPV18-positive), and Yumoto (HPV-negative) were significantly lower than that in non-cancerous cervical epithelium (P<0.0001; Fig. 1).

### Effects of miR-218 restoration on cell proliferation, migration and invasion in cervical SCC cell lines

**Figure 2:**
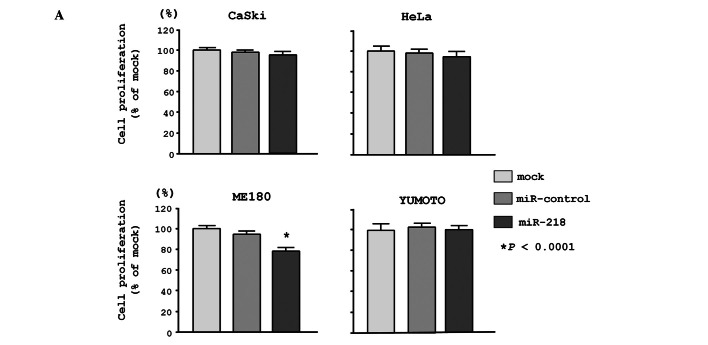

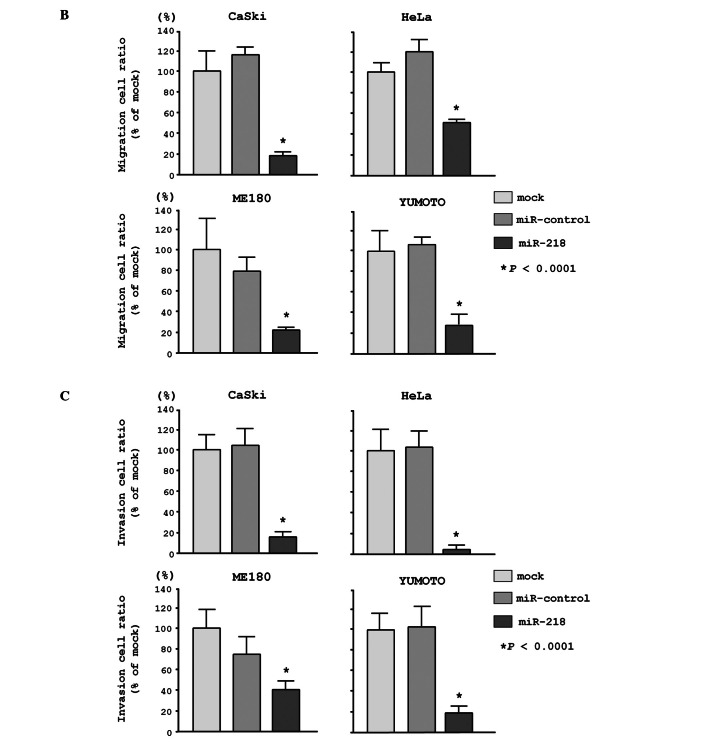
(A) Effects of *miR-218* restoration on proliferation of cervical-SCC cell lines. Proliferation activities of cervical-SCC cell lines (CaSki, ME180, HeLa and Yumoto) after transfection (72 h) with *miR-218* (10 nM) as determined by XTT assay. ^*^P<0.0001. (B) Effects of *miR-218* restoration on migration of cervical-SCC cell lines. Migration activities of cervical-SCC cell lines (CaSki, ME180, HeLa and Yumoto) after transfection (48 h) with *miR-218* (10 nM) as determined by migration assay. ^*^P<0.0001. (C) Effects of miR-218 restoration on invasion of cervical-SCC cell lines. Invasion activities of cervical-SCC cell lines (CaSki, ME180, HeLa and Yumoto) after transfection (48 h) with *miR-218* (10 nM) as determined by Matrigel invasion assay. ^*^P<0.0001.

To investigate the functional role of *miR-218*, we performed gain-of-function studies using cells transfected with a precursor of *miR-218*. The XTT assay revealed that cell proliferation was significantly inhibited in *miR-218* transfectants in comparison with non-transfectants (mock) and miRNA-control transfectants (control) in ME180 cells (78.1±3.7%, 100.0±2.5% and 96.0±3.5%, respectively; P<0.0001), while no significant inhibition was seen in CaSki cells (94.8±3.5%, 100.0±2.3% and 97.9±2.1%, respectively; P>0.0167), HeLa cells (93.0±5.0%, 100.0±7.5% and 97.6±4.1%, respectively; P>0.0167), and Yumoto cells (106.3±11.1%, 100.0±3.6% and 110.8±11.8%; P>0.0167; Fig. 2A).

Migration and Matrigel invasion assays demonstrated that the number of invading cells significantly decreased in *miR-218* transfectants in comparison with mock and miR-control transfectants in all cell lines tested. In fact, migration in *miR-218* transfectants was reduced to only 11.5±3.4% in CaSki cells (mock, 100.0±20.8; control, 115.4±12.2; P<0.0001), 20.3±3.8% in ME180 cells (mock, 100.0±28.1%; control, 77.2±13.8%; P<0.0001) 49.0±5.9% in HeLa cells (mock, 100.0±6.8%; control, 114.1±16.8%; P<0.0001), and 24.9±8.8% in Yumoto cells (mock, 100.0±18.2%; control, 102.2±6.8%; P<0.0001; Fig. 2B).

Similarly, in the Matrigel invasion assay, the number of invading cells was significantly decreased in *miR-218*-transfectants in comparison with mock and miR-control transfectants in all cell lines. Cell invasion was reduced to 3.9±1.6% in CaSki cells (mock, 100.0±8.5%; control, 107.0±16.3%; P<0.0001), 37.1±9.2% in ME180 cells (mock, 100.0±14.8%; control, 71.9±18.1%; P<0.0001), 2.7±1.5% in HeLa cells (mock, 100.0±21.6%; control, 102.7±19.0%; P<0.0001), and 14.9±6.6% for Yumoto cells (mock, 100.0±14.8%; control, 103.4±19.4%; P<0.0001; Fig. 2C).

### Identification of miR-218-mediated molecular pathways and putative miR-218 target genes

We first obtained putative *miR-218* target genes by searching the TargetScan database. According to the database, 4,946 conserved targets, with a total of 1,865 conserved sites and 4,372 poorly conserved sites, were deposited in this database. These genes were analyzed and characterized in KEGG pathway categories using the GeneCodis program. This analysis revealed 105 signaling pathways (Table III). In these pathways, we focused on the focal adhesion pathway and the 48 genes contained within this pathway (Table IV). To search for genes regulated by tumor-suppressive *miR-218* in cervical SCC, we applied gene expression profiles in the GEO database (accession no. GSE6791). Among 48 genes, 24 genes were upregulated in cervical SCC compared to adjacent non-cancerous tissues. The expression levels of up- or downregulated genes in clinical specimens are shown in Table IV. As a result of our expression data, we identified *LAMB3* as one of the most highly upregulated genes in clinical specimens; this gene has 1 putative *miR-218* binding site. *LAMB3* is a laminin that is an important and biologically active part of the basal lamina, functioning in a variety of pathways, such as cell differentiation, migration, adhesion, proliferation and survival. Thus, we focused on *LAMB3* as a promising target gene of *miR-218* in cervical SCC.

### Expression of LAMB3 in cervical SCC clinical specimens

**Figure 3:**
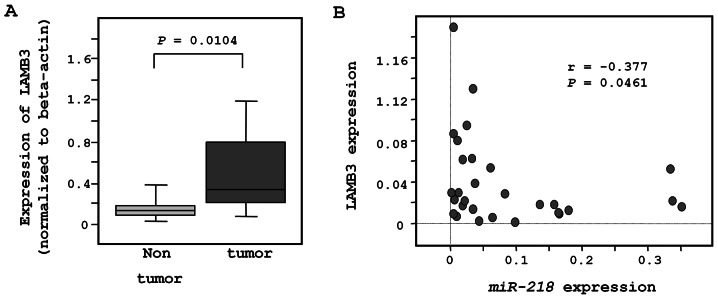
Expression of *LAMB3* in cervical-SCC clinical specimens. (A) Expression of *LAMB3* in cervical-SCC tissues and nontumor tissues as determined by qRT-PCR. *ACTB* was used as an internal control. (B) The correlation between the expression of *miR-218* and *LAMB3* was analyzed in cervical-SCC and non-tumor tissues.

The expression of *LAMB3* was significantly lower in clinical cervical SCC specimens (n=18; 0.053±0.049) than in non-cancerous specimens (n=11; 0.017±0.014, P=0.0104; Fig. 3A). Moreover, *LAMB3* expression was significantly inversely correlated with *miR-218* expression (r=−0.377; P=0.0461; Fig. 3B).

### LAMB3 is a direct target of miR-218

**Figure 4:**
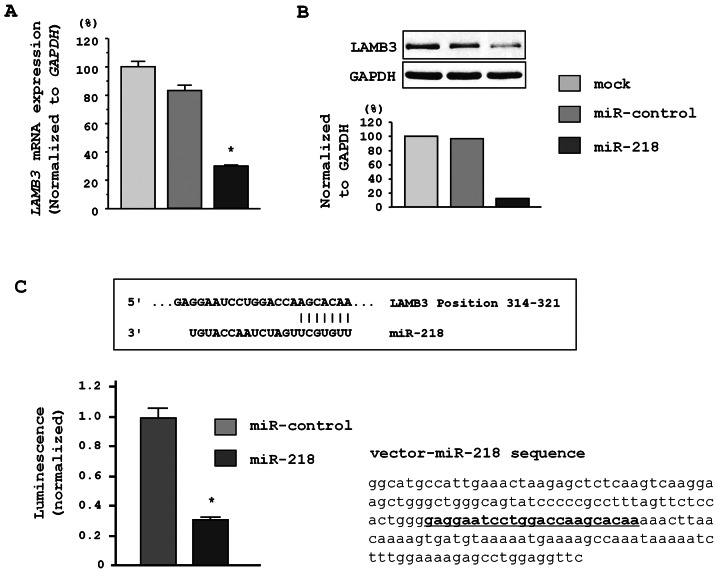
*miR-218* directly regulates *LAMB3*. (A) mRNA expression of *LAMB3* as measured by qRT-PCR 72 h after transfection with 10 nM *miR-218*. *GAPDH* was used as an internal control. ^*^P<0.0001. (B) Protein expression of LAMB3 as measured by western blot analysis 72 h after transfection with 10 nM *miR-218*. GAPDH was used as a loading control. The expression ratio of LAMB3 was evaluated using ImageJ software. (C) A putative *miR-218* binding site in the 3′UTR of *LAMB3* mRNA was identified using the TargetScan database (Upper). Luciferase reporter assays were performed using a vector encoding the partial sequences of the 3′UTR containing the putative *miR-218* target site. The vector (10 ng) and *miR-218* or *miR-control* (10 nM) were cotransfected into CaSki cells. Renila luciferase activity was measured 24 h after transfection. The results normalized by firefly luciferase values are shown. ^*^P<0.0001.

We performed quantitative real-time RT-PCR and western blot analysis to investigate whether *LAMB3* mRNA and protein were downregulated by restoration of *miR-218*. Importantly, both *LAMB3* mRNA and protein levels were significantly repressed in *miR-218*-transfectants in comparison with mock transfectants (Fig. 4A and B).

To determine whether the 3′UTR of *LAMB3* had an actual target site for *miR-218*, we performed a luciferase reporter assay by using a vector encoding the 3′UTR of *LAMB3* mRNA. We found that the luminescence intensity was significantly reduced in *miR-218* transfectants as compared to mock and miRNA-control transfectants (P<0.0001; Fig. 4C).

### Silencing of LAMB3 mRNA and protein in a cervical SCC cell line

**Figure 5:**
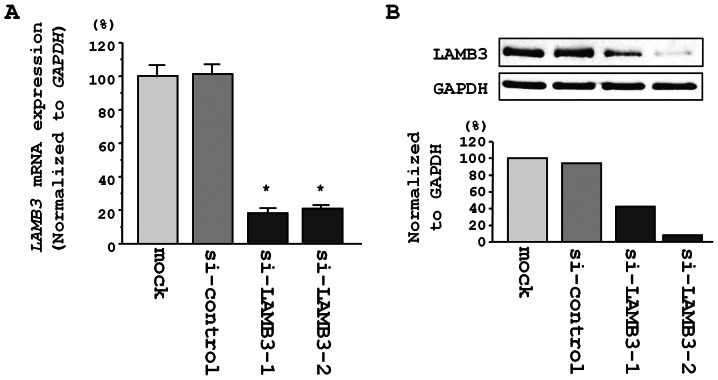
LAMB3 expression was suppressed by si-LAMB3 transfection at both the mRNA and protein levels in CaSKi cells. (A) mRNA expression of *LAMB3* as revealed by real-time qRT-PCR 72 h after transfection with 10 nM si-LAMB3. ^*^P<0.0001. (B) Protein expression of LAMB3 as revealed by western blot analysis 72 h after transfection with 10 nM si-LAMB3. GAPDH was used as a loading control. The expression ratio of LAMB3 was evaluated using ImageJ software.

Next, we examined the impact of si-*LAMB3* transfection in CaSki cells. The expression of *LAMB3* mRNA was reduced in 2 si-*LAMB3* transfectants in comparison with mock and si-control transfectants (P<0.0001; Fig. 5A). Additionally, the expression of LAMB3 protein was reduced in si-*LAMB3*-1 and si-*LAMB3*-2 transfectants in comparison with mock and si-control transfectants (P<0.0001 and P=0.0002, respectively; Fig. 5B). These results showed that the 2 siRNAs were useful for loss-of-function assays in this study.

### Effects of LAMB3 silencing on cell proliferation, migration and invasion in cervical SCC cell lines

**Figure 6:**
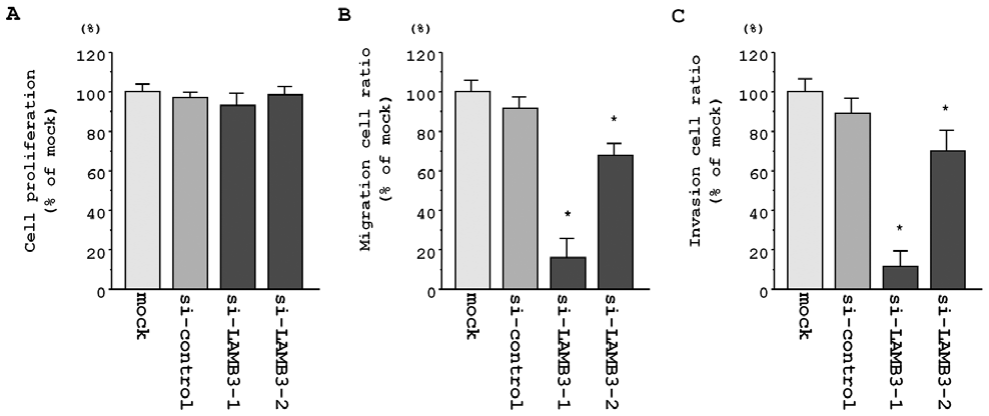
Effects of LAMB3 knockdown by si-LAMB3 transfection in CaSki cells. (A) Cell proliferation activities as revealed by XTT assay in the cervical-SCC cell line, CaSki. (B) Cell migration activities in CaSki cells. ^*^P<0.0001. (C) Cell invasion activities in CaSki cells. ^*^P<0.0001.

To investigate the functional role of *LAMB3*, we performed loss-of-function studies using si-*LAMB3* transfectants. The XTT assay revealed that cell proliferation was not inhibited in the 2 si-*LAMB3* transfectants as compared with mock and si-control transfectants in CaSki cells (82.4±10.7%, 100.2±6.1%, 100.0±14.6% and 95.6±11.2%; P>0.0083; Fig. 6A).

Additionally, the number of migrating cells was significantly decreased in both si-*LAMB3* transfectants as compared with mock and si-control transfectants in CaSki cells (10.6±2.7%, 72.2±10.7%, 100.0±6.5% and 91.8±11.0%, respectively; P<0.0001; Fig. 6B).

The number of invading cells was also significantly decreased in si-*LAMB3* transfectants as compared with mock and si-control transfectants in CaSki cells (13.5±4.9%, 73.1±16.2%, 100.0±13.7% and 88.4±18.0%, respectively; P<0.0001; Fig. 6C).

## Discussion

The discovery of non-coding RNA during the human genome sequencing project had a significant impact in cancer research (26). The reconstructing of genome-wide studies to include non-coding RNA is therefore necessary for cancer research. miRNAs are a class of small non-coding RNAs, and a growing body of evidence has suggested that miRNAs also contribute to cancer initiation, development and metastasis in many types of cancers, including cervical cancer (10).

It is believed that normal regulatory mechanisms can be disrupted by aberrant expression of tumor-suppressive or oncogenic miRNAs in cancer cells. Therefore, identification of aberrantly expressed miRNAs is the first step toward elucidating miRNA-mediated oncogenic pathways. Based on this, we identified the miRNA expression signatures in several human squamous cell carcinomas, including esophageal SCC, hypopharyngeal SCC, maxillary sinus SCC and lung SCC, allowing the elucidation of multiple tumor-suppressive miRNAs (12–15). Our previous studies showed that *miR-218* is a frequently downregulated miRNA and that restoration of this miRNA inhibited cancer cell migration and invasion in head and neck SCC (HNSCC) cells (19). We also searched for downregulated miRNAs in cervical SCC by examining expression signatures published in public databases (27–31). These data indicate that *miR-218* is frequently downregulated miRNA in cervical SCC. Thus, we focused on *miR-218* and investigated the functional significance of this miRNA in mediating cancer pathways.

In the human genome, 2 *miR-218* precursor genes, *miR-218-1* and *miR-218-2*, have identical sequences in the mature miRNA and map to human chromosomes 4p15.31 and 6q35.1, respectively. Interestingly, the genomic regions of *miR-218-1* and *miR-218-2* are located in the introns of the *SLIT2* and *SLIT3* genes, respectively. Downregulation of *miR-218* in cancer cells has been shown to be caused by promoter hypermethylation of *SLIT2* and *SLIT3* genes (20). Silencing of *miR-218* by DNA hypermethylation has also been reported in oral SCC using a function-based screening approach (21). On the other hand, several reports have indicated that silencing of *miR-218* in cervical SCC was caused by HPV infection (24,32). Our expression data showed that *miR-218* expression was significantly reduced in both HPV-positive cells (CaSki, HeLa and ME180) and HPV-negative cells (Yumoto), in addition to clinical specimens. Since the molecular mechanisms of *miR-218* silencing in cervical SCC are still unclear, further study is necessary to solve this problem.

In the current study, we found significant inhibition of cancer cell migration and invasion in cervical SCC cell lines (CaSki, HeLa, ME180 and Yumoto) by *miR-218* restoration, suggesting that *miR-218* is a tumor-suppressive miRNA in cervical SCC. Our previous reports in HNSCC also showed that *miR-218* contributes to cancer cell migration and invasion (19). The tumor-suppressive function of *miR-218* has also been reported in several types of cancers, and *miR-218* has been shown to target several oncogenic genes, such as Rictor (oral cancer), survivin and ROBO1 receptor (nasopharyngeal cancer and gastric cancer) (20–22). Our recent report also indicated that restoration of *miR-218* inhibited cancer cell proliferation, migration, and invasion in bladder cancer (23,33). These data suggested that *miR-218* is an important tumor-suppressive miRNA that is deeply involved in human cancers.

miRNAs are unique in their ability to regulate many protein-coding genes. Bioinformatic predictions have indicated that miRNAs regulate more than 30% of protein-coding genes (34). A single miRNA is capable of targeting a number of genes to globally regulate biological processes. The identification of novel cancer pathways and responsible genes regulated by tumor-suppressive *miR-218* in cervical SCC is the next step for our understanding of cervical SCC oncogenesis. Thus, we pursued GeneCodis analysis to reveal the functional significance of these genes potentially regulated by *miR-218* in cervical SCC. The GeneCodis analysis applies many genes to known pathways in the KEGG Pathway Database, and these data facilitate our understanding of tumor-suppressive miRNA-mediated molecular pathways in human cancer. This method of analysis has previously been used to efficiently identify tumor-suppressive miRNA-mediated cancer pathways in our laboratory (19). In the current study, the GeneCodis analysis revealed 105 signaling pathways, as highlighted in Table II. In these pathways, we focused on the focal adhesion pathway and the 48 genes contained within this pathway.

To search for genes regulated by tumor-suppressive *miR-218* in cervical SCC, we used gene expression profiling in this study (deposited in the GEO database as accession no. GSE6791). Among the 48 genes in the focal adhesion pathway, 10 were upregulated in cervical SCC clinical specimens, indicating that these genes were candidates for regulation by tumor-suppressive *miR-218* in cervical SCC. From these genes, we focused on *LAMB3*, a component of laminin-332, and investigated the functional significance of this gene.

Laminin-332, a heterotrimer composed of 3 chains (LAMA3, LAMB3 and LAMC2), is an adhesion substrate for epithelial cells and regulates epithelial cell migration during epithelial regeneration and repair processes (35,36). Several immunohistochemical studies have shown that laminin-332 or its subunit LAMC2 is expressed in tumor cells at the invasion front or in budding tumor cells in many types of human cancers, such as adenocarcinomas of the colon, breast, pancreas and lung, SCC of the esophagus and melanoma (35). Our data demonstrated that *LAMB*3 was directly regulated by *miR-218* and functioned as an oncogene, contributing to cancer cell migration and invasion in cervical SCC. Many studies have indicated that laminin-332 binds to several cell-surface receptors, such as integrins, epidermal growth factor receptor and syndecan-1 (37–39). Among these binding partners, integrins are cell surface transmembrane proteins that mediate extracellular signals and intracellular pathways, leading to control of the cell cycle, cell migration, and invasion in cancer cells (40). It will be necessary to analyze the signal pathways associated with the interactions of integrins and laminin-332 in cervical SCC in the future.

## Figures and Tables

**Table I:** Characteristics of cervical-SCC specimens and non-cancerous specimens.

No.	Age	FIGO stage	Tumor size (cm)	Lymph node metastasis	HPV status

1	58	IIB	1.7×1.9	−	16
2	64	IIB	ND	−	16
3	37	IIB	3.5×3.0	+	16
4	41	IB2	8.3×3.3	−	16
5	39	IB1	3.5×3.4	−	16
6	34	IB1	3.2×2.2	−	16
7	43	IB2	4.0×8.0	−	18
8	56	IIIB	3.0×3.1	+	16,18
9	77	IIB	3.0×2.7	−	16
10	62	IB1	3.0×2.0	−	16
11	56	IIIA	4.5×2.2	+	16
12	56	IIA	4.0×4.0	−	16
13	60	IB1	4.0×4.0	−	16
14	32	IIB	6.0×3.0	+	16
15	38	IB2	6.8×4.6	+	16
16	44	IB1	3.5×2.2	−	16
17	40	IB1	3.0×2.0	−	16
18	63	IB1	2.7×2.4	−	16

No.	Age	HPV status			

1	44	-			
2	77	-			
3	75	-			
4	45	-			
5	47	-			
6	69	-			
7	40	-			
8	48	-			
9	41	-			
10	41	-			
11	34	-			

ND, no data.

**Table II:** Primer sequences of HPV detection.

Primer	Sequence (5′ to 3′)	Location	Product size (bp)
HPV 16 E6-F	GCACCAAAAGAGAACTGCAATGTT	85–108	142
HPV 16 E6-R	AGTCATATACCTCACGTCGCAGTA	203–226	
HPV 16 E7-F	CAAGTGTGACTCTACGCTTCGG	738–759	81
HPV 16 E7-R	GTGCCCATTAACAGGTCTTCCAA	796–818	
HPV 18 E6-F	CTATAGAGGCCAGTGCCATTCG	503–524	79
HPV 18 E6-R	TTATACTTGTGTTTCTCTGCGTCG	558–581	
HPV 18 E7-F	TAATCATCAACATTTACCAGCCCG	721–744	113
HPV 18 E7-R	CGTCTGCTGAGCTTTCTACTACTA	810–833	

**Table III:** Significantly enriched annotations regulated by *miR-218 (*top 20 pathways).

No. of genes	P-value	Annotations
59	1.55E-16	Endocytosis
40	4.29E-12	Glutamatergic synapse
69	4.30E-11	Pathways in cancer
58	4.21E-10	MAPK signaling pathway
38	4.87E-10	Insulin signaling pathway
48	4.98E-10	Focal adhesion
29	1.53E-09	ErbB signaling pathway
25	3.70E-09	Long-term depression
35	6.58E-09	Axon guidance
43	1.99E-08	Chemokine signaling pathway
24	6.10E-08	Chronic myeloid leukemia
23	6.35E-08	Long-term potentiation
36	1.00E-07	Wnt signaling pathway
33	1.06E-07	Tight junction
26	1.21E-07	Prostate cancer
26	1.21E-07	Gap junction
21	2.87E-07	Glioma
26	2.98E-07	FcγR-mediated phagocytosis
22	5.37E-07	Adherens junction
38	5.54E-07	Calcium signaling pathway

**Table IV:** Expression of target genes by *miR-218* involved in focal adhesion pathways.

Entrez gene	Gene symbol	FC	Regulation	P-value
3265	HRAS	4.54	Up	4.19E-02
3914	LAMB3	3.54	Up	4.40E-03
3676	ITGA4	3.21	Up	6.03E-03
5062	PAK2	2.40	Up	7.29E-05
1282	COL4A1	2.33	Up	2.21E-02
6464	SHC1	2.15	Up	3.05E-04
87	ACTN1	2.14	Up	3.18E-03
1399	CRKL	2.10	Up	4.50E-04
2932	GSK3B	1.83	Up	1.36E-03
394	ARHGAP5	1.75	Up	9.48E-04
3915	LAMC1	1.75	Up	8.18E-03
3371	TNC	1.51	Up	3.09E-01
5501	PPP1CC	1.50	Up	6.03E-03
387	RHOA	1.45	Up	2.04E-01
1301	COL11A1	1.44	Up	3.87E-01
5829	PXN	1.33	Up	1.27E-01
858	CAV2	1.27	Up	3.09E-01
3480	IGF1R	1.26	Up	2.42E-01
100291393	LOC100291393	1.09	Up	8.39E-01
25759	LOC100291393	1.09	Up	8.39E-01
2321	FLT1	1.05	Up	7.22E-01
5601	MAPK9	1.03	Up	8.39E-01
896	CCND3	1.02	Up	8.79E-01
5293	PIK3CD	1.02	Up	8.39E-01
3691	ITGB4	−1.02	Down	9.59E-01
5500	PPP1CB	−1.07	Down	4.76E-01
63923	TNN	−1.17	Down	4.46E-01
1289	COL5A1	−1.17	Down	6.84E-01
5170	PDPK1	−1.19	Down	7.51E-02
5058	PAK1	−1.23	Down	1.40E-01
7057	THBS1	−1.23	Down	4.46E-01
10451	VAV3	−1.23	Down	7.60E-01
5156	PDGFRA	−1.41	Down	2.63E-01
10000	AKT3	−1.46	Down	6.71E-02
1956	EGFR	−1.51	Down	5.99E-02
399694	SHC4	−1.57	Down	9.33E-02
55742	PARVA	−1.58	Down	1.68E-02
5579	PRKCB	−1.71	Down	9.50E-03
53358	SHC3	−1.80	Down	5.33E-02
5649	RELN	−1.81	Down	2.52E-02
2318	FLNC	−1.81	Down	2.21E-02
5295	PIK3R1	−1.88	Down	4.19E-02
1277	COL1A1	−2.38	Down	8.38E-02
8515	ITGA10	−2.61	Down	9.01E-05
6714	SRC	−2.85	Down	1.36E-03
81	ACTN4	−2.86	Down	3.05E-04
3479	IGF1	−2.97	Down	8.18E-03
595	CCND1	−5.52	Down	1.14E-03
